# Cystic Fibrosis-Related Diabetes in Poland

**DOI:** 10.3390/ijerph19074069

**Published:** 2022-03-29

**Authors:** Marta Rachel, Marek Biesiadecki, Sabina Galiniak

**Affiliations:** 1 Institute of Medical Sciences, Medical College, Rzeszów University, Warzywna 1a, 35-310 Rzeszów, Poland; mbiesiadecki@ur.edu.pl (M.B.); sgaliniak@ur.edu.pl (S.G.); 2 Department of Allergology and Cystic Fibrosis, State Hospital 2 in Rzeszow, Lwowska 60, 35-301 Rzeszów, Poland

**Keywords:** diabetes, cystic fibrosis-related diabetes, F508del mutation, prevalence

## Abstract

Cystic fibrosis (CF) is the most common autosomal recessive inherited monogenic disease in Caucasians. As medical technology progresses and the quality of patient care improves, the survival time of patients with CF has increased, which results in more frequent comorbidities such as cystic fibrosis-related diabetes (CFRD). CFRD is the result of abnormal glucose metabolism characterized primarily by insulin deficiency, exacerbated periodically by insulin resistance. The aim of our study was to analyze the epidemiology of patients with CFRD in Poland on the basis of data collected from six CF treatment centers. Analyses were performed on 1157 CF patients who were treated at one of the six CF care centers. CFRD was diagnosed according to standard criteria. All data including demographics, types of CFTR mutations, CFRD duration, and microorganisms in the sputum were obtained from the patients’ medical history. Our study indicates that the prevalence of CFRD in Poland is 12.9%. CFRD was most often diagnosed between the ages of 11 and 20 (60% of patients), while 23% of patients were diagnosed between 21 and 30 years of age. Furthermore, we observed that approximately 3–5% of patients under the age of 10 had CFRD. We found out that the type of mutation did not affect the frequency of CFRD development. Factors that increased the risk of developing CFRD include underweight and chronic *Pseudomonas aeruginosa* infection. Due to the extended lifespan of CF patients, the number of CFRD patients is currently increasing. We believe that the results of our study may complement information from other studies or may be useful in planning health policy in Poland.

## 1. Introduction

Cystic fibrosis (CF) is one of the common chronic diseases inherited in an autosomal recessive inheritance pattern, with mean incidence ranging from 1:1353 in Ireland to 1:10,000 in Russia [1]. CF is caused by mutations in the CF transmembrane conductance regulator (CFTR) gene, which encodes the CFTR chloride-anion channel expressed in several tissues. As recently as fifty years ago, about 90% of infants with CF died before the age of two. Currently, it is estimated that a patient born in the last two decades of the 20th century in an economically developed country has an over 50% chance of reaching ca. 40 years of age [2,3,4]. Along with the prolonged survival of patients, the number and importance of diseases related to CF have increased [5,6,7]. One such complication is diabetes mellitus, which is referred to as cystic fibrosis-related diabetes (CFRD) and might appear at any age. CFRD prevalence increased by approximately ten percentage points every decade from ten years of age [8]. CFRD is a unique disease that combines features of patients with type 1 and type 2 diabetes mellitus. The primary etiology is relative insulin insufficiency secondary to destruction of pancreatic islets [9,10]. It is difficult to predict which patients will develop the disease; however, some mutations in CFTR have a higher prevalence and predisposition to develop the disease and are associated with pancreatic exocrine dysfunction [8]. Risk factors for CFRD include female gender, age, liver disease, and pancreatic insufficiency [11,12,13]. Moreover, CFRD is associated with a decline in lung and liver functions, impairments in weight gain and growth, impairment of pubertal development, depression, and increased morbidity and mortality [8,9,10,11,12,13,14,15]. The 2010 clinical care guidelines for CFRD recommend insulin as the mainstay of treatment. However, many patients with CFRD may not require exogenous insulin due to the heterogeneity of this clinical entity [16]. Therefore, the aim of the study was to analyze and present the characteristics of patients with CFRD collected on the basis of data from six CF treatment centers in Poland.

## 2. Materials and Methods

### 2.1. Ethical Considerations

All procedures applied during the study were in accordance with the ethical standards of the responsible committee on human experimentation and with the Helsinki Declaration of 1975 as revised in 2008. Informed consent for being included in the study was obtained from all patients. The study was approved by the local Bioethical Commission of the University of Rzeszów (9 January 2020).

### 2.2. Population

Analyses were performed on 1157 CF patients who were evaluated at one of the 6 participating sites (Gdańsk, Poznań, Warsaw, Lublin, Rabka-Zdrój, Katowice) between 1 January 2018 and 31 January 2020. The presence of at least one clinical symptom of the disease, a positive family history (sick siblings), or a positive CF newborn screening test (patients born in or after 2009) resulted in a suspicion of CF. All patients had confirmed diagnosis of CF by sweat chloride test ≥60 mmol/L (gold standard) and CFTR gene mutations due to the fact that genetic testing for CF in newborn babies is not routinely performed in Poland. CFRD was diagnosed based on symptoms according to standard American Diabetes Association criteria (fasting plasma glucose levels ≥126 mg/dL (7.0 mmol/L) on two or more occasions, 2 h postprandial plasma glucose levels ≥200 mg/dL (11.1 mmol/L) persisting for more than 48 h, HbA_1c_ ≥ 6.5%, classic symptoms of diabetes—polyuria and polydipsia) [16,17,18]. At the same time, differential diagnosis was performed, and thyroid diseases were excluded. All CFRD patients were treated with insulin as recommended by a diabetologist according to blood glucose levels.

**Figure 1:**
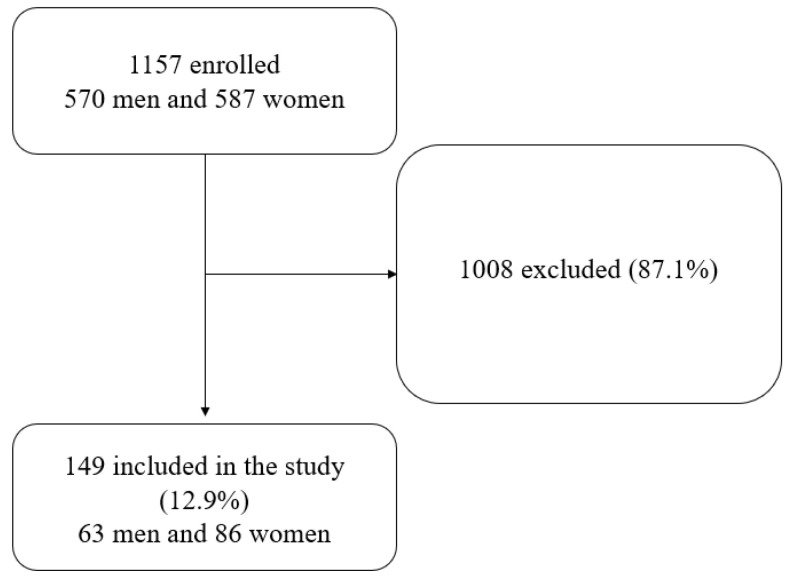
Flowchart of the study recruitment process.

A flow chart outlining the recruitment process is shown in Figure 1. Among the studied CF patients, there were 149 individuals who had CFRD, while 1008 patients were excluded from the study due to the lack of CFRD.

All data including demographics (age, gender, age at CF diagnosis), body mass index (BMI), genetic screening for CFTR mutations (F508del, G542X, N1303K, and other), CFRD duration, and chronic infection with microorganisms in the sputum (*Pseudomonas aeruginosa, Staphylococcus aureus*, and *Aspergillus fumigatus*) were obtained from the patients’ medical history. Patient therapy in centers was carried out according to the recommendations [16]. The CFRD evaluation was outside of the exacerbation periods.

### 2.3. Statistical Analysis

The results were processed using the SPSS software (IBM, SPSS Statistics, Armonk, NY, USA). For descriptive analysis, mean and standard deviation (SD) were used for continuous variables, while absolute frequency (percentage) was used for categorical variables. Normality of distribution was validated using the Shapiro–Wilk test and skewness and kurtosis values, as well as visual assessment of histograms. Fisher’s exact test and the χ2 test were performed to compare the groups using categorical variables. Differences between groups were evaluated using nonparametric tests (Mann–Whitney U test). All tests were two-tailed with α = 0.05. Furthermore, hazard ratios were used to identify significant independent predictors of CFRD. The hazard ratios calculated from our own observations were compared with the data obtained from the ECFS Patient Registry [19].

## 3. Results

Overall, these health centers treated 149 CF patients diagnosed with CFRD (63 male and 86 female subjects). The sex distribution of the entire population was 42.3% males and 57.7% females. However, female patients were slightly more affected by CFRD at a prevalence of 14.6% compared with 11% in male subjects. Patient characteristics are summarized in Table 1.

**Figure 2:**
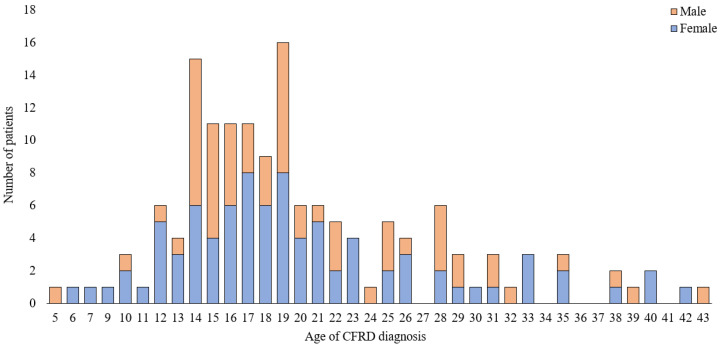
Age of CFRD diagnosis by sex group.

There were no differences in the mean patient age or the age at CF and CFRD diagnosis between male and female participants. Most of CFRD patients were between 17 and 25 years old at the time of the study. Figure 2 presents the exact age of patients at CFRD diagnosis by sex group. The analysis showed that CFRD in both female and male patients was most often diagnosed between the ages of 11 and 20 (60% of patients), while 23% of patients were diagnosed between 21 and 30 years of age.

Nevertheless, we found that CFRD duration was longer in female than in male subjects (*p* < 0.05). In addition, no differences were observed in BMI values at the time of the study as well as at the time of CFRD diagnosis between the studied female and male participants. Among adult participants, low bodyweight (BMI < 18.5) was observed in 20 women (35.1%) and 9 men (26.4%) at the moment of CFRD diagnosis. However, at the moment of the study, BMI was lower than 18.5 in 10 women (18.9%) and 6 men (12.2%). In the pediatric population, 9 female (31%) and 11 male (38%) participants had low bodyweight (BMI, Z-score <−2) at the time of CFRD diagnosis. On the other hand, at the time of this study, underweight was noted in six females (42.8%) and seven males (50%). BMI at the time of CFRD diagnosis did not increase when compared with BMI calculated at the time of the study in the pediatric and adult population (*p* = 0.644 and *p* = 0.263). Sweat chloride concentration was similar between female and male patients. Among the study groups, approximately 90% of patients used pancreatic enzymes, and 20% had liver function impairment. Three patients had a lung transplant. A total of 42 patients (28.2%) had hepatomegaly. The patients’ respiratory function expressed as forced expiratory volume in one second (FEV_1_) was between 23% and 70%.

**Figure 3:**
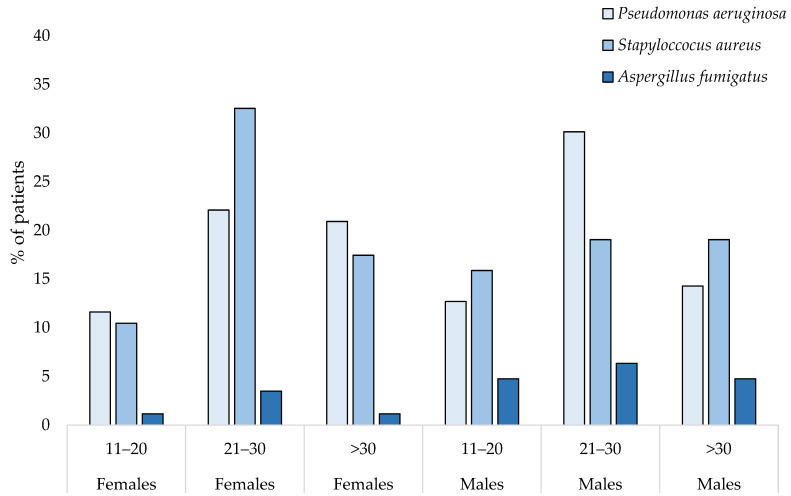
Percentage of chronic infections in CFRD patients by sex and age group.

There was no difference in the number of patients chronically infected with *P. aeruginosa* and *S. aureus* between females and males. However, we noticed that more males with CFRD were infected with *A. fumigatus* (*p* < 0.05) than female patients. Sixteen female participants with CFRD were co-infected with *P. aeruginosa* and *S. aureus*, while there were eight co-infected patients among male subjects. The largest group of infected patients ranged between 21 and 30 years of age (25.5% of patients with *P. aeruginosa* and 26.8% with *S. aureus*). Figure 3 presents the percentage of chronic infections among CFRD patients by age and sex group.

The most common CF mutation F508del/F508del was present in 44.9% of the CFRD patients (*n* = 67). Consequently, 31.5% of patients had the F508del/other genotype, while 23.6% of participants had the other/other genotype.

Table 2 presents hazard ratios of genetics, BMI, and chronic bacterial infections for CFRD. According to the results, the type of mutation did not affect the frequency of CFRD development.

Our results prove that underweight patients had an almost 5 times greater chance of developing CFRD; on the other hand, an optimum or excess weight alleviated the risk of developing CFRD. Likewise, chronic *P. aeruginosa* infection caused a 4 times greater risk of developing CFRD, while the *S. aureus* infection was not associated with an increased risk of developing CFRD.

## 4. Discussion

Glucose metabolism in CF is variable and depends on the patient’s clinical condition, nutrition, respiratory infections, and increased energy expenditure. Generally, it is estimated that 70% to 90% of people with CF will have been diagnosed with one type of diabetes by the age of 40 [20]. Evidently, patients with CFRD have a significantly reduced health-related quality of life, and their diagnosis and treatment require the coordinated work of specialists from various fields of medicine [21,22]. Moreover, they have difficulties with higher-level processes known as “executive function”, which demand greater cognitive load and recruit the prefrontal cortex [23]. CFRD is associated with poor prognosis in individuals with CF, while early diagnosis and aggressive treatment contribute to improvement in survival [24,25]. Additionally, pubertal stage in patients with CFRD was more delayed than in controls [26]. According to the study by Olsen et al., the prevalence of CFRD varies by country: from 3% in Ukraine to as high as 31.9% in Denmark [8]. In our study, the prevalence of CFRD in Poland was 12.9%, which may be defined as average for the purpose of the CFRD prevalence study in different countries [24]. Nevertheless, Fendler et al. noted the increase in the prevalence of CFRD in Poland between 2005 and 2011, which could be a result of the improved care of CF children but also an increased awareness of the disease’s diabetogenic potential and reduced patient mortality [3,27]. Similarly, the Cystic Fibrosis Foundation Patient Registry, which collects data from the United States, reported that the diagnosis of CFRD is becoming more frequent. In 1997, about 5–6% of patients with CF were diagnosed with CFRD, while in 2013 that ratio was already 30%. In the 1990s, the mean age of patients at the diagnosis of CFRD was between 18 and 21 years [28]. This average age at diagnosis continues today. According to recommendations, annual screening for CFRD should start by the age of 10 in all CF patients because clinical deterioration in nutritional and pulmonary status begins from ca. half a year to two years prior to the CFRD diagnosis [14,29]. However, according to our research, approximately 3–5% of CFRD cases begin earlier, even as early as at the age of 5. Similarly, CFRD was also reported in children with CF <6 years old [30]. According to the data of the European Epidemiologic Registry of Cystic Fibrosis, the incidence of CFRD is 5% in patients aged 10–14 and 13% in patients aged 15–19 [31]. Similarly, the study of Soave et al. noted that the risk of developing CFRD may be predicted in early life and that worse exocrine pancreatic disease in infancy predicts CFRD at an older age [32].

In our own research as well as in the research of other authors, the prevalence of CFRD in the female sex is noticeable [8,11,33]. A study by Lewis et al. proved that CFRD prevalence was higher at every age in females than males [34]. CFRD may reduce the patient’s weight, increasing the risk of death in CF patients. Many authors confirm that weight loss and BMI occur several years before the diagnosis of CFRD [35,36]. In our adult population, BMI at the diagnosis of CFRD was 19.2 ± 3.4 kg/m^2^. Recently, BMI has slightly increased to 19.9 ± 3 kg/m^2^, which may indicate that patients are adhering to dietary recommendations and that the quality of medical care is satisfactory. A recent study indicates that patients with CFRD had lower growth velocity and reduced weight gain rate compared with patients without CFRD between 5 and 10 years [37]. At the same time, the Cystic Fibrosis Genetic Analysis Consortium records over 2100 mutations in the CFTR gene, whereas 66% of all mutations are F508del [38]. F508del/F508del was also the most common mutation in the CFRD patients analyzed for this study. Similar results were obtained by Laubner et al. and Street et al. [39,40]. Several studies reported that impaired glucose metabolism belongs to a common comorbidity in both homozygous and heterozygous subjects with the F508del mutation [41]. Abnormal glucose levels in CF patients may increase the risk of bacterial colonization. In the first years of life, the most frequently isolated pathogen is *S. aureus*; it is estimated that this infection affects over 60% of children with CF. The incidence of infections caused by *P. aeruginosa* increases with age. Among patients diagnosed with CFRD, the type of bacteria does not differ significantly from those with CF alone. However, in the study by Cawood et al. colonization with *P. aeruginosa* bacteria in CFRD patients was 82.5% among the studied patients, compared with 64.2% of non-diabetic CF patients [42]. Likewise, study of Olsen et al. presented that the most common colonization was bacteria from the *Pseudomonas* and *Burkholderia* groups among CFRD patients [8]. They also noted the increase in the number of chronic infections among CFRD patients with age. In our study, the decline in the number of infections after the age of 30 may result from the lower number of patients in this age group due to the mortality of patients and the fact that the average lifespan of CF patients in Poland is 24.5 ± 8.9 years [3].

Summarizing, according to recommendations, patients with CFRD should be screened annually beginning five years after the diagnosis of CFRD for diabetic complications, as prolonged life expectancy and improvement in clinical condition may increase the incidence of microvascular complications [17]. Research indicates that approximately 30% of CFRD patients have microvascular complications including microalbuminuria, retinopathy, neuropathy, and nephropathy [43,44]. Moreover, in recent years, more and more attention has been paid to the impact of the wide spectrum of glucose metabolism derangements in patients affected by CF. Research indicates that glucose metabolism derangements are very common in children with CF with pancreatic insufficiency, even under the age of 6 years, including infants [30,45,46]. Screening methods used in the general population can also be used in patients with CF; it should be noted, however, that their condition associated with the course of the underlying disease may interfere with the specificity of the markers. It is worth noting that Lin et al. proposed an application that allows the estimation of an individual’s CFRD risk at different ages over the course of their disease, which may be helpful for physicians who deal with the daily care of CF patients [24].

The data on patients with a diagnosed CFRD in Poland analyzed in this study are not complete. They come from six CF treatment centers. In Poland, there are no accurate epidemiological data due to the lack of a nationwide registry kept for these patients. Moreover, Polish data were often missing in international registries.

## 5. Conclusions

Due to the extended lifespan of CF patients in Poland, the number of adults and adolescents is increasing. In conclusion, the study shows that in Poland women with CF develop CFRD more often and faster. Additionally, two factors (underweight and chronic bacterial infection with *P. aeruginosa*) increased the chance of developing CFRD. It is worth noting that approximately 5% of children with CF under the age of 10 years develop CFRD. As there are few data on CFRD in Poland, the results of our study will certainly increase knowledge on the complications of CF and its epidemiology, as well as informing health policy planning.

## Figures and Tables

**Table 1:** Demographic characteristics of patients with CFRD at the time of the study and at the diagnosis of CFRD; *p* value was determined by Mann–Whitney U test *.

	Male	Female	*p*
Number of patients	63	86	
Age	26.5 ± 8.3 (12–49)	27.8 ± 8.5 (11–51)	0.382
Age at CF diagnosis (years)	6.8 ± 7.4 (1–39)	7.2 ± 7.5 (1–40)	0.881
Age at CFRD diagnosis (years)	20.2 ± 7.5 (5–43)	19.9 ± 7.6 (6–41)	0.894
CFRD duration (years)	6.4 ± 4.5 (1–22)	8.2 ± 5.5 (2–23)	0.048
BMI (Z-score), patients <20 years	−1.83 ± 1.39 (−4.91–0.48)	−1.53 ± 1.96 (−6.91–1.08)	0.367
BMI (kg/m^2^), patients >20 years	19.5 ± 2.7 (14–28)	20.1 ± 3 (13–31)	0.38
BMI at CFRD diagnosis (Z-score), patients <20 years	−1.75 ± 1.48 (−5.74–0.53)	−1.14 ± 1.47 (−4.45–1.29)	0.143
BMI at CFRD diagnosis (kg/m^2^), patients >20 years	19.6 ± 3.1 (14–27)	19.3 ± 3.7 (13–28)	0.476
Sweat chloride concentration (mmol/L)	114.6 ± 18.9 (70–147)	114.3 ± 19 (83–175)	0.665
Any use of pancreatic enzyme, n (%)	56 (89%)	80 (93%)	
Any hepatic dysfunction, n (%)	14 (22%)	17 (20%)	
Former organ transplantation, n (%)	1 (1.6%)	2 (2.3%)	

* Data are presented as mean ± SD (range).

**Table 2:** Hazard ratios of CFRD for genetics, BMI, and bacteria *.

	ECFS Patient Registry	Own Research	Hazard Ratio	Comment
CFTR mutation				
F508del/F508del	0.686	0.67	1.17	Similar chances
F508del/other	0.739	0.689	0.93	Similar chances
Other/other	0.202	0.236	0.98	Similar chances
BMI				
Underweight	0.118	0.568	4.81	Almost 5 times higher chance
Optimum	6.061	1.614	0.27	More than 4 times less chance
Overweight	0.147	0.021	0.14	More than 7 times less chance
Chronic infection				
*P. aeruginosa*	0.2913	1.273	4.37	More than 4 times higher chance
*S. aureus*	1.3913	1.381	0.99	Similar chances

* The table is based on own research and ECFS Patient Registry.

## Data Availability

Data supporting the results of this study shall, upon appropriate request, be available from the corresponding author.
